# Validation of the optical Aktiia bracelet in different body positions for the persistent monitoring of blood pressure

**DOI:** 10.1038/s41598-021-99294-w

**Published:** 2021-10-19

**Authors:** Josep Sola, Anna Vybornova, Sibylle Fallet, Erietta Polychronopoulou, Arlene Wurzner-Ghajarzadeh, Gregoire Wuerzner

**Affiliations:** 1 Aktiia SA, Rue du Bassin 8a, 2000 Neuchâtel, Switzerland; 2 Service of Nephrology and Hypertension, CHUV - Lausanne University Hospital and University of Lausanne, Lausanne, Switzerland

**Keywords:** Cardiology, Hypertension

## Abstract

The diagnosis of hypertension and the adjustment of antihypertensive drugs are evolving from isolated measurements performed at the physician offices to the full phenotyping of patients in real-life conditions. Indeed, the strongest predictor of cardiovascular risk comes from night measurements. The aim of this study was to demonstrate that a wearable device (the Aktiia Bracelet) can accurately estimate BP in the most common body positions of daily life and thus become a candidate solution for the BP phenotyping of patients. We recruited 91 patients with BP ranging from low to hypertensive levels and compared BP values from the Aktiia Bracelet against auscultatory reference values for 4 weeks according to an extended ISO 81060-2 protocol. After initializing on day one, the observed means and standard deviations of differences for systolic BP were of 0.46 ± 7.75 mmHg in the sitting position, − 2.44 ± 10.15 mmHg in the lying, − 3.02 ± 6.10 mmHg in the sitting with the device on the lap, and − 0.62 ± 12.51 mmHg in the standing position. Differences for diastolic BP readings were respectively of 0.39 ± 6.86 mmHg, − 1.93 ± 7.65 mmHg, − 4.22 ± 6.56 mmHg and − 4.85 ± 9.11 mmHg. This study demonstrates that a wearable device can accurately estimate BP in the most common body positions compared to auscultation, although precision varies across positions. While wearable persistent BP monitors have the potential to facilitate the identification of individual BP phenotypes at scale, their prognostic value for cardiovascular events and its association with target organ damage will need cross-sectional and longitudinal studies. Deploying this technology at a community level may be also useful to drive public health interventions against the epidemy of hypertension.

## Introduction

Blood pressure (BP) is a strong predictor of cardiovascular risk when it is measured accurately and in a standardized way^1^. However, epidemiologic data show that prediction of risk is largely improved when BP is measured out of the doctor’s office. Accordingly, the latest international guidelines recommend more widespread use of out-of-office BP measurements for the diagnosis and follow-up of hypertension^1–3^.

Today, out-of-office measurements of BP are mostly performed by a series of self-measurements done at home and for long periods: the so-called Home Blood Pressure Monitoring (HBPM) method. Alternatively, patients may wear a device that repeatedly measures BP for 24 h: the so-called Ambulatory Blood Pressure Monitoring (ABPM) method^4^.

With both monitoring methods, different patterns of abnormal BP variability have been described in the last decades: namely white-coat hypertension, masked hypertension, nocturnal non-dipping, nocturnal extreme-dipping, nocturnal rising, nocturnal hypertension or high BP variability^5^. A growing body of evidence shows that such abnormal BP phenotypes are associated with an increase in cardiovascular events when compared to normal circadian rhythms, independently of BP readings from the physician office^6^. Based on this data, the universal large-scale cardiovascular risk assessment based on BP phenotyping should be the next step in the fight against hypertension and its associated cardiovascular disease. However, the current HBPM/ABPM available devices still rely on a technology that restricts out-of-office BP measurements, can be uncomfortable and sparse and is finally relatively expensive and only accessible to a limited fraction of the population^7^. Several authors have described clinical utilities of these office and out-of-office BP measurement methods and are combined and scored in Table 1^8–13^.

**Table 1:**
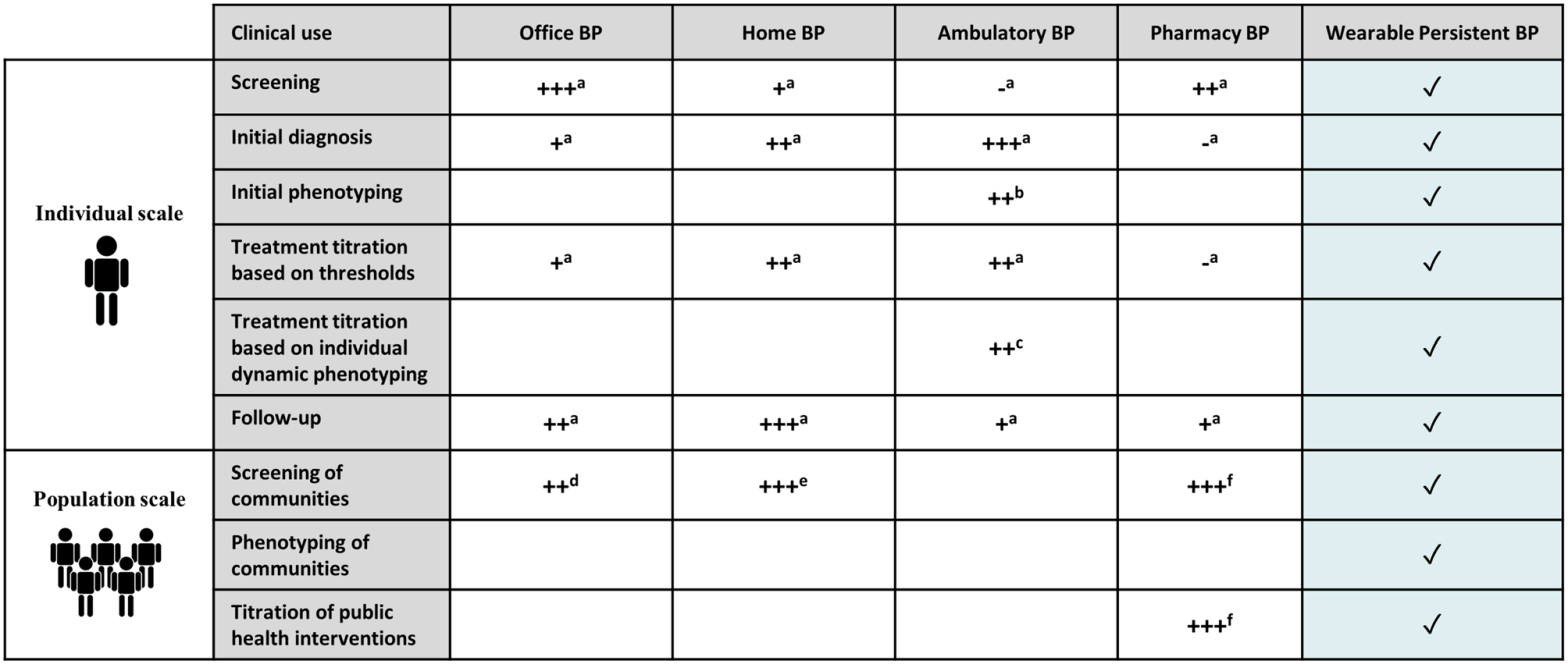
Scores on the potential use and utility of office BP, home BP, ambulatory BP and pharmacy BP taken from^8^ (marked by^a^), ^9^ (marked by^b^), ^10^ (marked by^c^), ^11^ (marked by^d^), ^12^ (marked by^e^) and ^13^ (marked by^f^).

In the digital era, wearable devices have already started to measure basic cardiovascular parameters. Empowered by optical sensors embedded on smartwatches and smart bands, a large catalogue of cardiovascular indicators can now be measured continuously, non-invasively and unnoticed by the wearer. The basic parameters that can be monitored by these devices range from heart rate, heart rate variability, detection of atrial fibrillation, SpO_2_, actigraphy to sleep patterns^14^. However, reliable, and validated devices measuring BP in different body positions and repeatedly over 24 h are unavailable so far.

The potential impact of widespread monitoring of BP through the optical sensors embedded on wearable devices is extensive: a 24/7 BP device would be an excellent candidate to not only provide an alternative to HBPM/ABPM in terms of cost-efficiency and patient adherence but also to extend existing use-cases by providing insights into individual daily, monthly and yearly BP rhythms. This would enable the first-ever large-scale phenotyping of populations in addition to driving personalized approaches for the diagnosis and the management of hypertension^15,16^. Table 1 marks with ✓ the uses and utilities that wearable persistent BP monitors have the potential to support.

**Figure 1:**
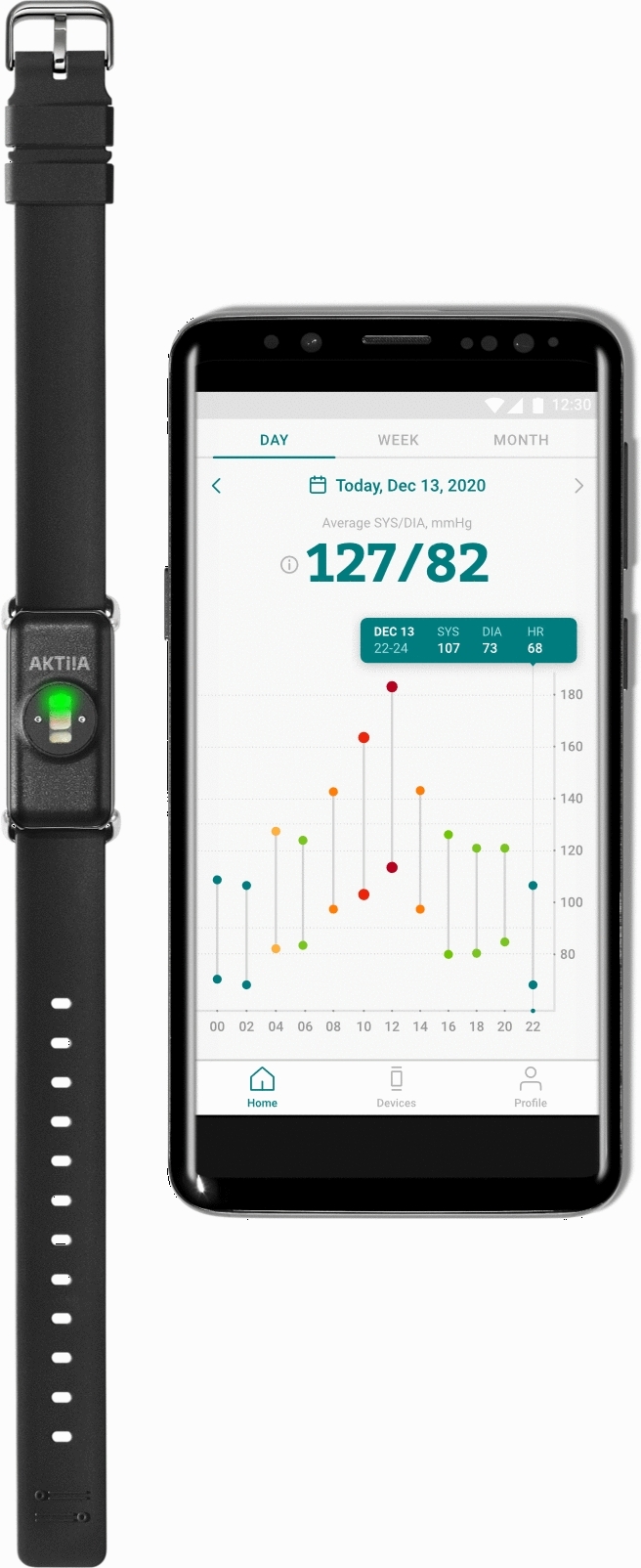
The Aktiia 24/7 BP monitor is a wearable device that persistently measures optical signals on the skin vasculature of the wrist and displays day and night BP values on a companion smartphone application. The Aktiia Bracelet is a CE-marked class IIa medical device.

The Aktiia Bracelet is a wearable CE-marked Class lla medical device (Fig. 1) that records optical signals at the wrist and transforms them into the BP values using pulse wave analysis algorithms after an initial calibration procedure based on a series of oscillometric measurement performed by an upper arm cuff^17^. The device and technology have already been validated according to international standards against invasive arterial line measurements in the lying position in ICU settings^18^, and against auscultation measurements in the sitting position^19^. However, the potential of this device goes beyond on-demand measurements when a patient is sitting or lying. Indeed, the Aktiia Bracelet allows automatic BP measurements in any body and arm positions as long as the arm is still, removing thus the need of the user to initiate a measurement and enabling thus persistent day and night BP monitoring.

In this paper, we report the performance of the optical Aktiia Bracelet to that of manual double-blinded auscultation measurements in a representative cohort of participants measured in different body positions. As such, we provide the first evidence of the feasibility of persistent BP monitoring via optical sensors at the wrist. Following these results, we propose a possible framework for the large-scale deployment of BP phenotyping of populations based on wrist-worn wearable devices that integrate optical sensors, and we outline the possible implications that these devices might have on global awareness and control of high BP.

## Methods

### Ethical consent and study registration

The study^20^ was approved by the local Ethics Committee (CER-VD—Commission cantonale d’éthique de la recherche sur l’être humain, CH 1012 Lausanne, Switzerland, authorization no. 2019-01046 of 06/08/2019) and the Swiss authority responsible for the authorization and supervision of therapeutic products (Swissmedic, CH-3012 Bern, Switzerland, authorization no. 10000542 of 29/07/2019). The study was registered in EUDAMED (registration no. CIV-19-06-029004 of 29/07/2019) and ClinicalTrials.gov (registration no. NCT04027777 of 22/07/2019).

All study participants signed an informed consent form prior to study procedures. The study was conducted according to the current version of the World Medical Association Declaration of Helsinki, ICH-GCP guidelines and ISO 14155. No adverse event was reported during this clinical investigation.

### Study objectives and outcomes

The main objective of the study was to demonstrate the accuracy and precision of BP values estimated by the Aktiia Bracelet when compared to BP readings obtained by manual double-auscultatory in the most common body positions of daily life. The study outcomes were the mean and the standard deviation of the differences of the readings of the two measurement modalities in each body position.

### Study population

The study population included a representative cohort of male and female patients (N = 91) aged between 21 and 65 years old (38 ± 13 years), with body mass index (BMI) between 18 and 41 kg/m^2^ (24.0 ± 4.7 kg/m^2^) and presenting different skin pigmentations and different hair follicle densities. The cohort covered all BP categories ranging from hypotensive to hypertensive as required by ISO 81060-2:2013 except for the requirement that 20% of readings shall have SBP ≥ 140 mmHg: this deviation resulted from a multi-factorial recruitment process that included, on top of the inclusion criteria of ISO 81060-2:2013, additional constraints on skin pigmentation, wrist hair follicle density, and age distribution. Yet, the recruited cohort represented all relevant BP phenotypes, including 24% of the patients presenting either SBP ≥ 140 mmHg or DBP ≥ 100 mmHg (hypertensive phenotype), and 24% of the patients presenting either SBP ≤ 100 mmHg or DBP ≤ 60 mmHg (hypotensive phenotype). Note that 91 patients were recruited instead of 85 (as required by ISO81060-22,013) in order to fulfil the described multi-factorial recruitment process. Patients with a wrist circumference larger than 22 cm were exclude from the study because the Aktiia Bracelet would not fit their wrist. Full details on the study recruitment matrix are available in a previous publication^19^.

### Experimental set-up

**Figure 2:**
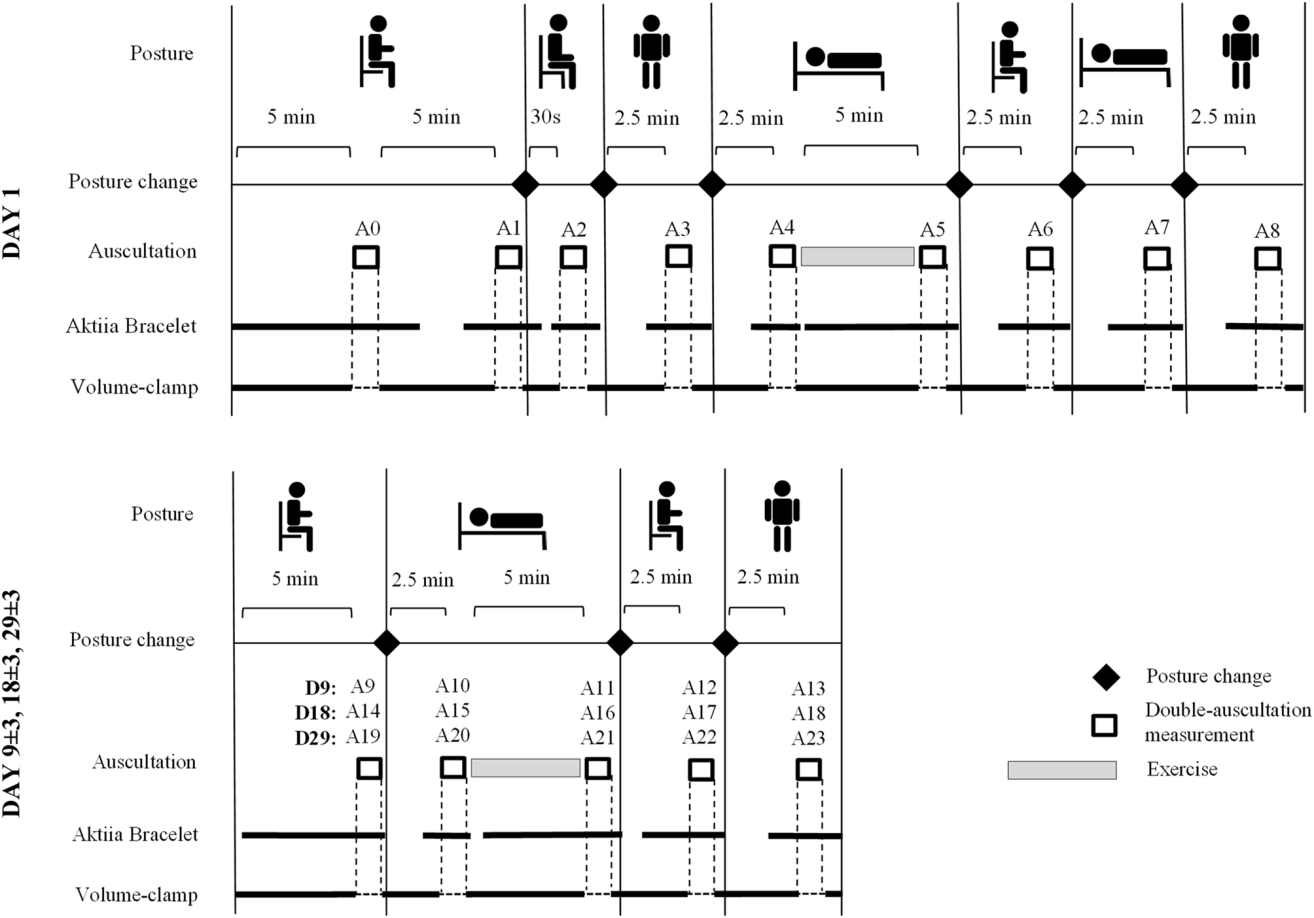
Schematic representation of the protocol designed to evaluate the performance of a cuffless BP monitor intended to be used in 24/7 environments. At each visit, assessments involving the reference device and the device under test are performed in varying body positions such as sitting, lying and standing positions, and after exercise. Visits are further repeated in order to test the stability of the device.

The study was held at the Lausanne University Hospital (Switzerland) and consisted of 4 visits over 1 month, i.e. at days 1, 9 ± 3, 18 ± 3, and 29 ± 3, involving a series of assessments in different body positions (sitting with de device at the heart level, sitting with the device on the lap, lying and standing) at each visit (Fig. 2). Each study participant was equipped with a manual sphygmomanometer on the left upper arm, and an Aktiia Bracelet on the right wrist. Each assessment of the study consisted of the simultaneous recording of optical signals with the Aktiia Bracelet and the reading of reference BP values by double-auscultation. As described and explained previously^19^, this was based on the requirements of the opposite limb simultaneous method described in ISO 81060-2:2013, excluding the use of alternating arms. The recording of an optical signal consisted of the acquisition of 30 s of green reflective photo-plethysmography^21^ at 100 samples per second. The reading of a reference BP value consisted of the identification of the K1 and K5 Korotkoff sounds by two experienced observers during the manual deflation of the sphygmomanometer cuff. Full disclosure of the implemented measuring methods is provided in^19^.

**Figure 3:**
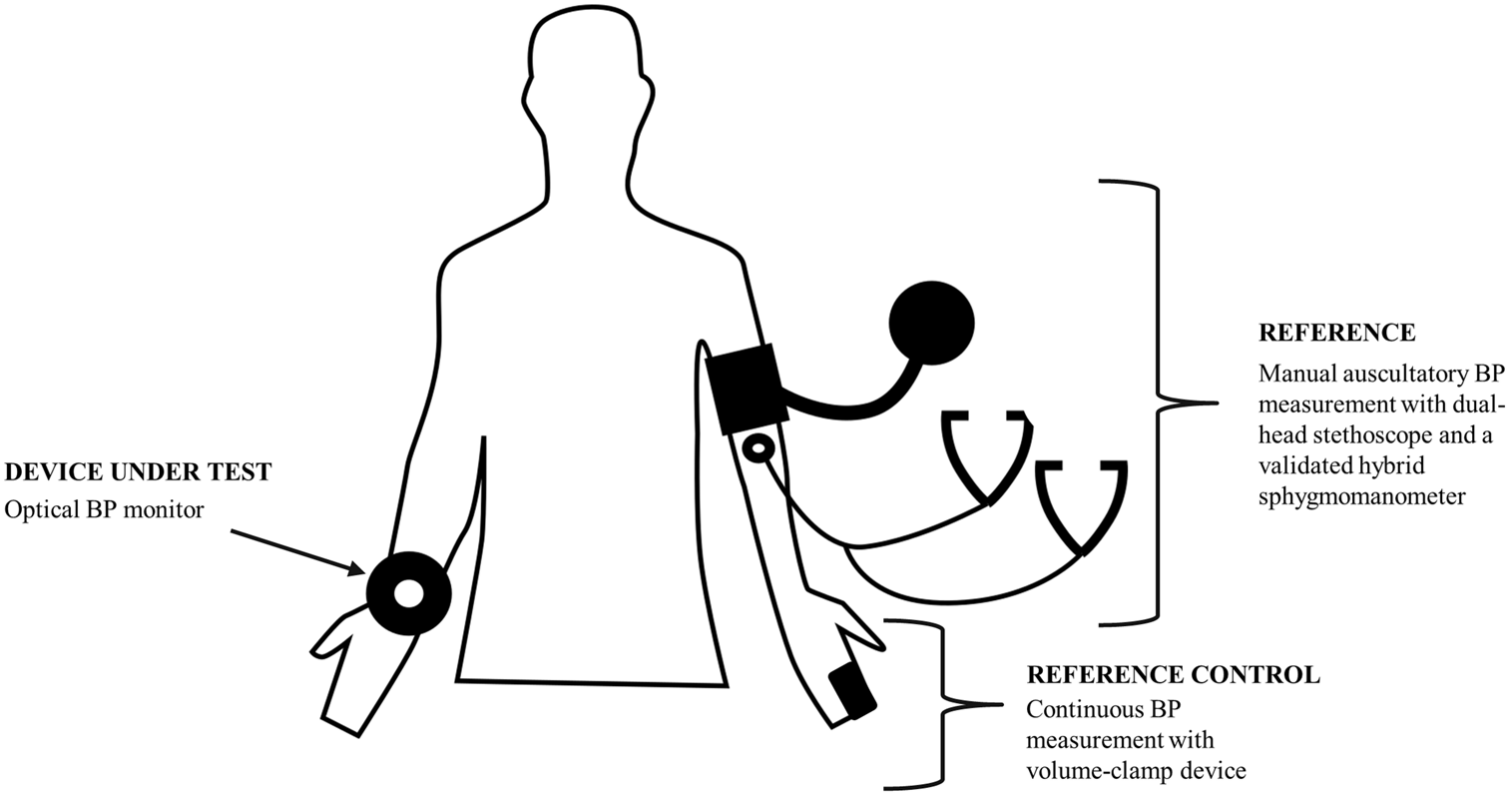
Experimental set up of the required equipment to investigate the performance of a BP monitor intended to be used in 24/7 environments. Reference BP values are obtained by a validated hybrid sphygmomanometer and auscultation with a dual-head stethoscope. Changes in auscultation values with different body positions are controlled by the continuous measurements of an ipso-lateral volume-clamp device. Measurements by the device under test are performed in the contra-lateral arm.

Participants were also equipped with a volume-clamp device on the left arm to be used as a reference-control device for changes in BP induced by different body positions (Nexfin BMYE, Netherlands). For this purpose, a finger cuff of appropriate size was positioned on the participant’s left middle finger and continuously monitored finger arterial pressure throughout the entire duration of the visit (Fig. 3).

### Measurement procedures in addition to sitting and relaxed conditions

To assess the performances of the Aktiia Bracelet on the realistic ambulatory scenario of BP monitoring around the clock, the protocol included BP assessments in different body postures: sitting and relaxed, lying supine and standing (Fig. 2). A 5-min aerobic exercise was also performed at each visit to assess the ability of the Aktiia Bracelet to capture large BP changes. In addition, and to assess the effect of the arm position related to the heart level in the performance of the device, measurements with the arm on the lap (i.e. below the heart level) were also performed. Each BP assessment in each described condition consisted of a simultaneous recording of optical signals with the Aktiia Bracelet, a BP measurement using double-auscultation, and a BP measurement using volume-clamp.

### Limits of the auscultatory technique as a reference method

When used in patients that are “seated and relaxed”, double auscultation is widely used as a reference method for the validation of BP monitors. However, the goal of the present study was to assess the performances of a wearable device when used in 24/7 conditions, including positions other than sitting (lying and standing) and after physical activity (aerobic exercise). Our group has recently published experimental data^22^ suggesting that the reliability of auscultation in other conditions than such “seated and relaxed” can be compromised if the vascular tonus of the brachial artery is altered. To mitigate the uncertainty introduced by double auscultation in such non-standard measurement conditions, the current study introduced an additional comparator intended to verify the accuracy of the reference double-blinded auscultatory measurements: the finger volume-clamp measurement device^23^ (Nexfin, BMEYE BV, The Netherlands). Volume-clamp has been repeatedly been shown to be unaffected by vasomotion effects induced by body position changes and physical exercise^24–26^ and appears to be a more reliable reference BP monitoring method under such conditions. It is important to note that the current study introduced volume-clamp to assess the reliability of a BP trend as seen by auscultation at each episode of a body position change (*i.e.* assessing the difference between two consecutive measurements), but was not used to assess the accuracy of BP readings (*i.e.* comparing absolute values of BP from both measurement modalities).

### Data analysis

#### Reference-control of auscultation readings by volume-clamp

**Figure 4:**
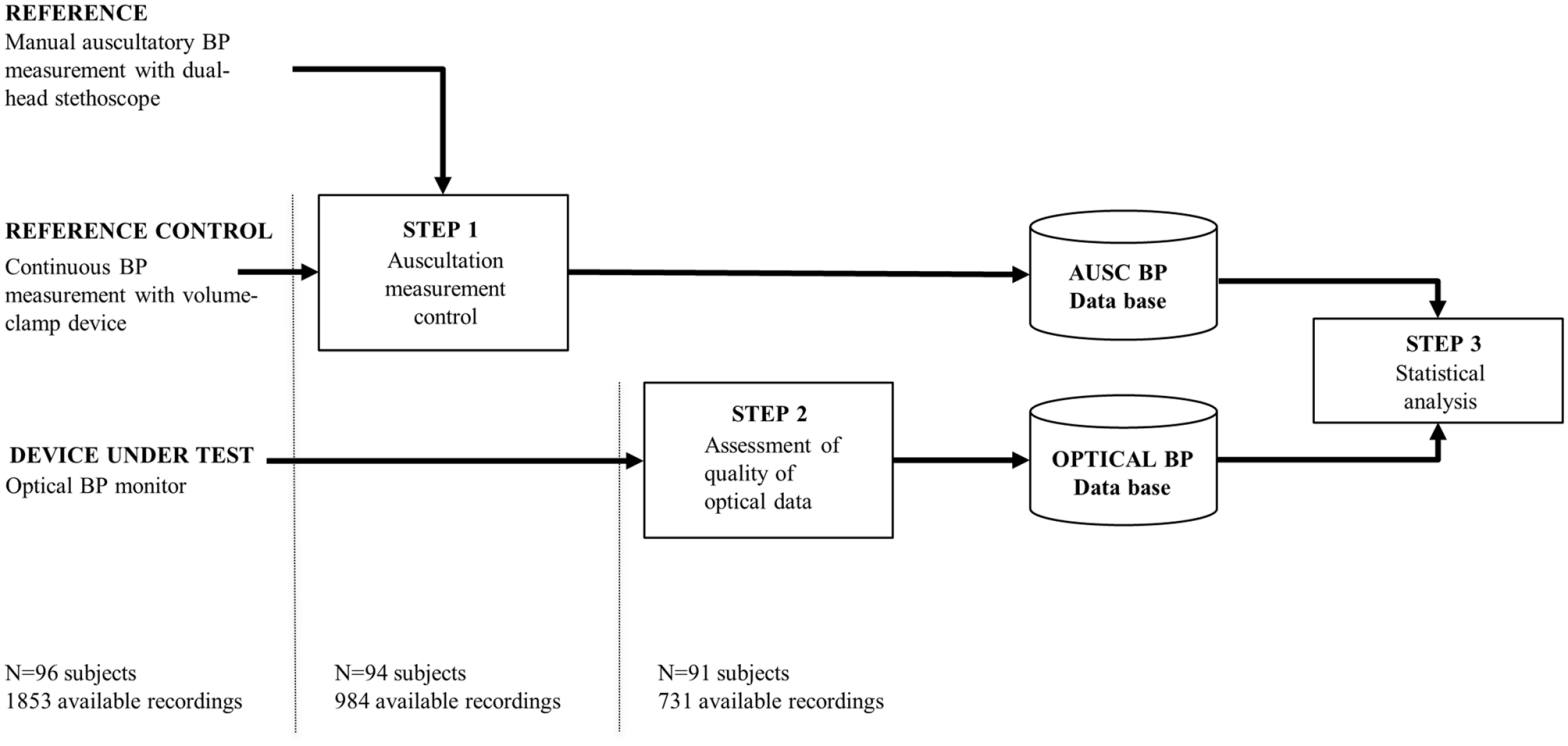
Implemented data analysis controlling the reliability of auscultatory BP measurements by means of a reference control (volume-clamp measurements), and further identifying assessments for which quality of optical signals is sufficient to execute Pulse Wave Analysis routines of the algorithms of Aktiia Bracelet. The number of available subjects and recording at each step is reported.

For each assessment, an auscultation reference value was computed as the average value between the readings of the two observers (following the guidance of ISO81060-2^27^ as described in^19^). Furthermore, for those assessments that were not performed in the standard sitting-setting and relaxed conditions, the reliability of auscultation readings was controlled using a volume clamp. The reference-control was performed as follows: if a BP change as measured by the two modalities (auscultation versus volume-clamp) was detected in agreement (in the same direction) and differed less than 10 mmHg, the auscultatory BP reference value was retained for further analysis. Otherwise, the reliability of auscultation was considered unsatisfactory, and the assessment was rejected. This reference-control procedure is illustrated in Step 1 of Fig. 4. Note that volume-clamp BP readings are not further treated as an independent set of reference values for this study: their use is restricted to the reference-control process described in this section.

#### From the optical signals to calibrated BP values

For each assessment that was retained for further analysis, the raw optical signal recorded by the Aktiia Bracelet was analyzed by Aktiia algorithms to generate one uncalibrated SBP value and one uncalibrated DBP value. The generation of uncalibrated BP values was performed based on the principle of Pulse Wave Analysis as described in^28,29^. Assessments with optical signals depicting insufficient quality (due to movement artifacts or reduced skin perfusion) were automatically discarded by Aktiia algorithms and not retained for further analysis as described in^29^ (Fig. 4, step 2). Finally, uncalibrated SBP and DBP values were transformed into calibrated BP values as described in^19^.

### Statistical analysis

To evaluate the ability of the Aktiia Bracelet to generate BP values for measurement conditions occurring in real-life scenarios, the percentage of assessments for which the recorded optical signals could be analyzed by the Aktiia algorithms was calculated for each measurement condition (acceptance rate index).

To provide performance figures of the Aktiia Bracelet when used in a sitting and relaxed condition, reference auscultatory values were compared against Aktiia readings according to criterion 1 and criterion 2 of ISO 81060-2 (as reported in^19^). These are the same criteria used in the validation of automated oscillometric cuffs in the same standardized sitting measurement conditions. For all the other measurement conditions, performance figures of the Aktiia Bracelet were calculated as the mean and the standard deviation of the differences between Aktiia Bracelet readings and auscultatory values (no pass/fail criterion from ISO81060-2 is applicable for these non-standard measurement conditions).

Scatter plots, correlation coefficients and Bland Altman plots comparing reference auscultatory values against Aktiia readings were generated for all measurement conditions following the guidelines of AAMI/ESH/ISO^30^.

## Results

After performing the data processing steps described in Fig. 4, a total of 731 paired BP measurements from 91 participants were retained for further analysis.

**Figure 5:**
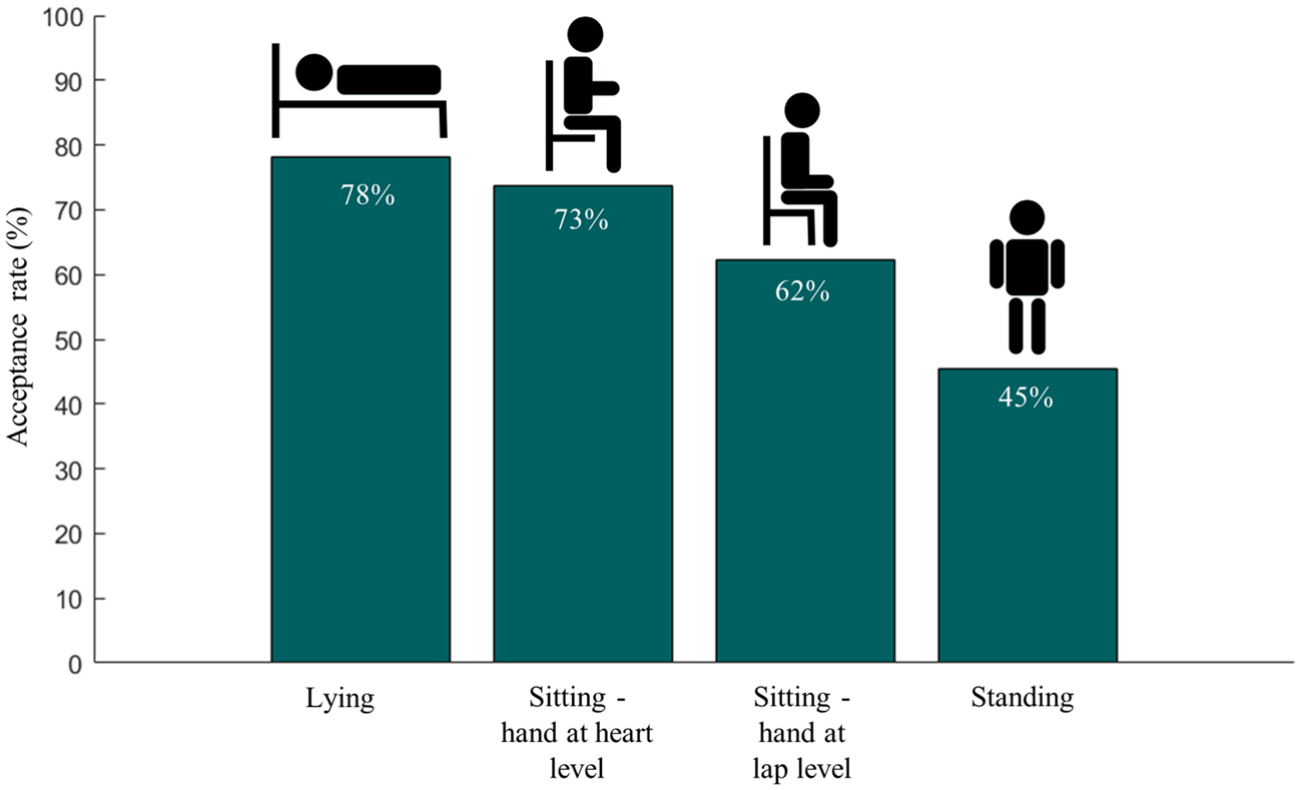
Acceptance rate of the Pulse Wave Analysis algorithms of Aktiia bracelet for each body position: percentage of time for which the recorded optical signals could be analyzed to generate a SBP and a DBP value.

The acceptance rates (percentages of retained measurements) for each measurement condition are reported in Fig. 5. For lying and sitting positions, the Aktiia Bracelet could generate BP estimates from the optical signals more than 70% of the time. When the wrist equipped with the bracelet was lowered to the lap level in the sitting position (around 50 cm below heart level), the acceptance rate decreased to 62%. The lowest acceptance rate was observed on standing position (45%).

For all measurement conditions, the mean and standard deviation of the differences between the two measurement modalities are reported in Table 2. In terms of precision, the lying and standing positions were associated with the largest standard deviations for both systolic and diastolic BP readings. It is important to highlight the fact that the bias between both methods (measured as the average of the differences) did not significantly change when moving the wrist from the level of the heart to the level of the lap level in the sitting position. As already reported in^19^, for the sitting and relaxed position for which ISO81060-2 performance criteria are applicable, the obtained results meet criterion 1 and criterion 2 of the standard.

**Table 2:** Performance of the Aktiia Bracelet when compared against double auscultation for different body positions.

	N readings	Systolic blood pressure			Diastolic blood pressure		
		Accuracy	ISO81060-2		Accuracy	ISO81060-2	
		Mean ± Std (mmHg)	Criterion 1	Criterion 2	Mean ± Std (mmHg)	Criterion 1	Criterion 2
Sitting—wrist at heart level	335	0.46 ± 7.75	PASS	PASS	0.39 ± 6.86	PASS	PASS
Sitting—wrist at lap level	177	− 3.02 ± 6.10	N/A	N/A	− 4.22 ± 6.56	N/A	N/A
Lying	146	− 2.44 ± 10.15	N/A	N/A	− 1.93 ± 7.65	N/A	N/A
Standing	73	− 0.62 ± 12.51	N/A	N/A	− 4.85 ± 9.11	N/A	N/A
All positions	731	− 1.11 ± 9.85	N/A	N/A	− 1.32 ± 7.56	N/A	N/A

Results depict mean and standard deviation of the differences between the two measuring modalities, and pass/fail criteria of ISO81060-2 when applicable^19^.

**Figure 6:**
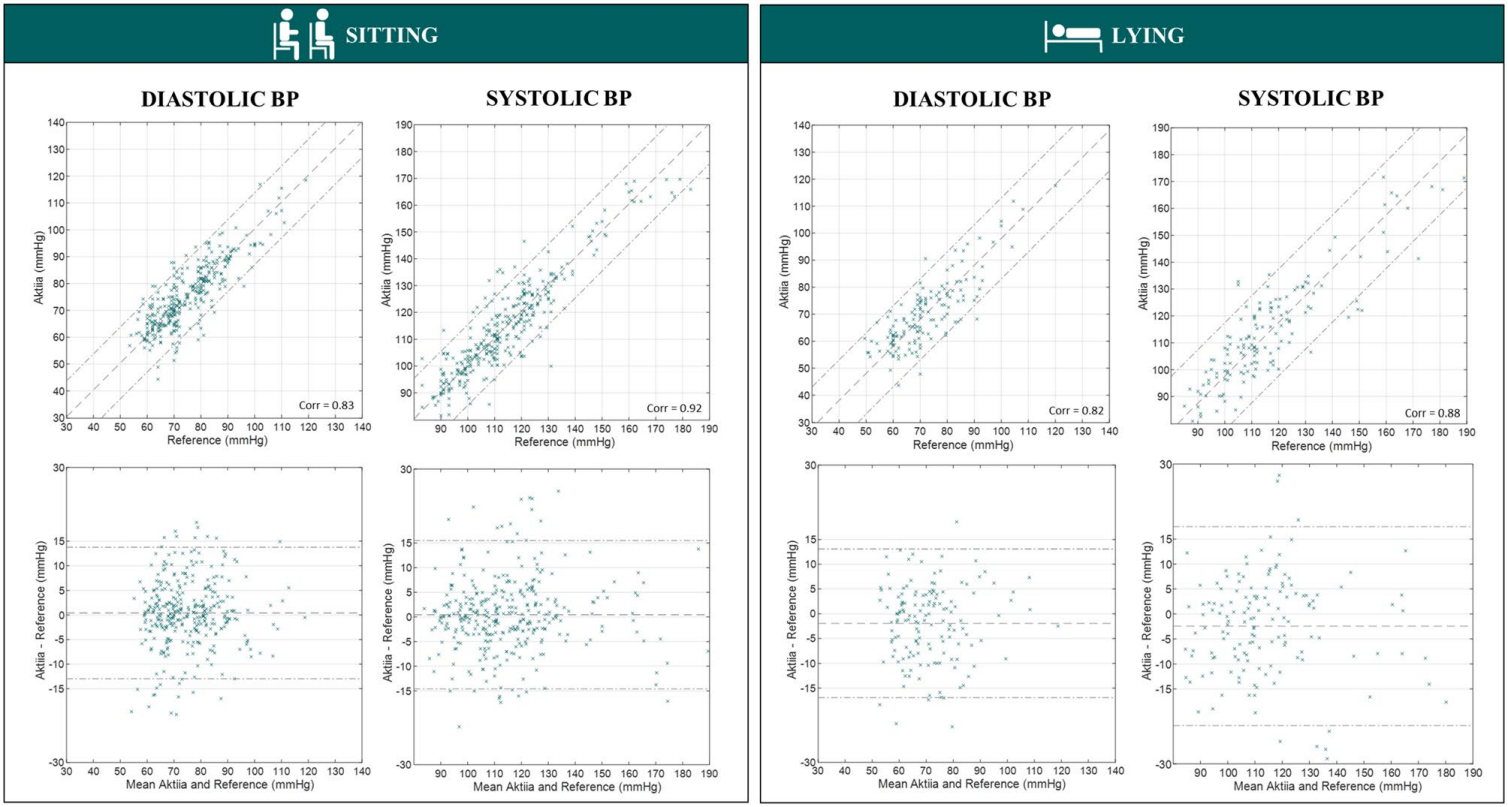
Scatter plots and Bland Altman plots comparing Aktiia BP readings against reference auscultatory readings for sitting and lying positions.

**Figure 7:**
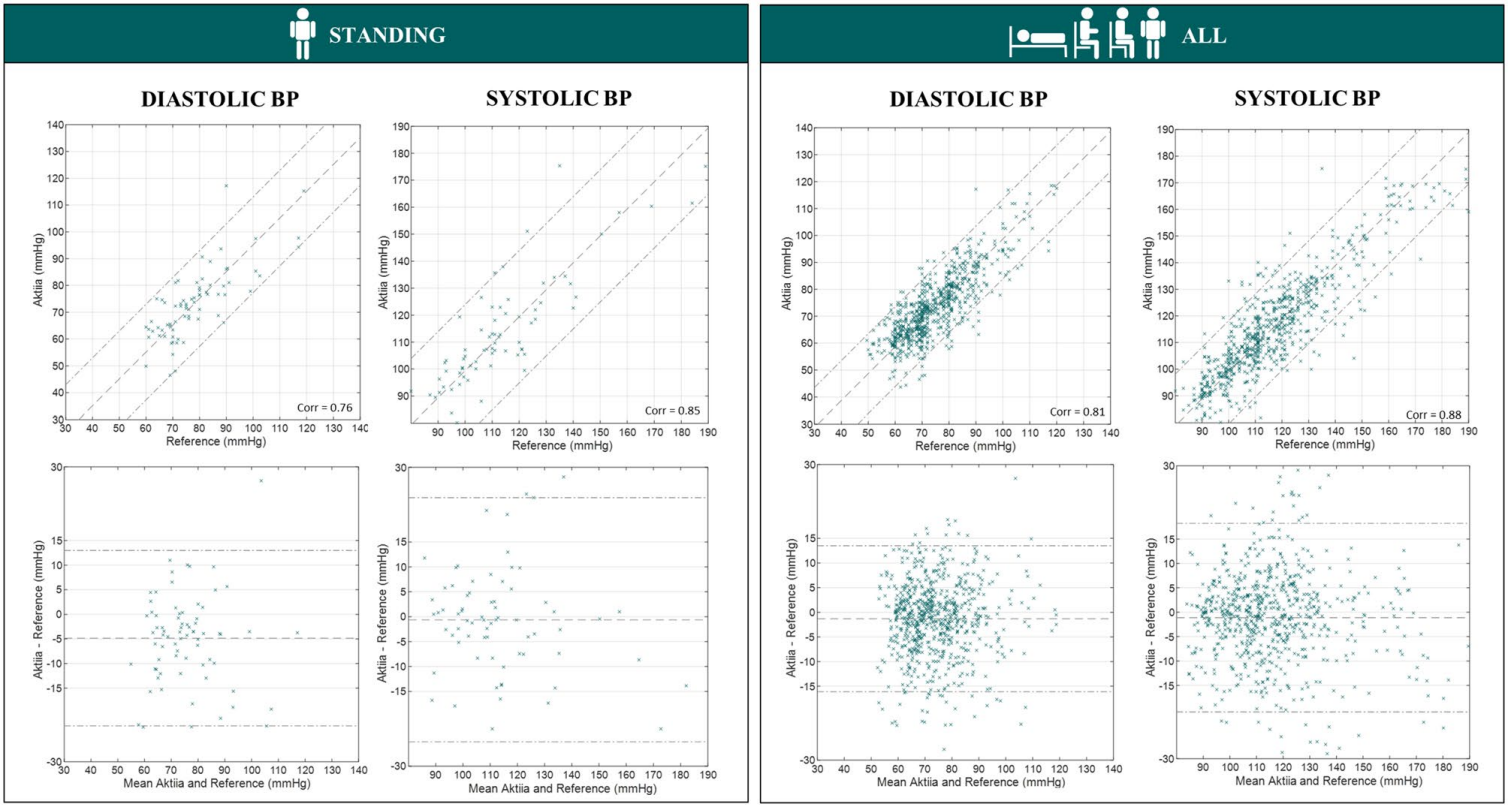
Scatter plots and Bland Altman plots comparing Aktiia BP readings against reference auscultatory readings for standing and all positions.

Finally, the scatter plots and standardized Bland–Altman plots for SBP and DBP for all measurement conditions are illustrated in Figs. 6 and 7.

The participants reported that BP measurement with the Aktiia Bracelet was unnoticed and rather enjoyable compared to the traditional auscultatory method.

## Discussion

In this study, we demonstrated that the Aktiia bracelet is capable of generating BP readings across different motionless conditions, with high probabilities of generating readings in sitting and lying positions. In a sitting position, when the wrist is lowered to the lap level, a decreased acceptance rate is observed most likely due to a reduced arterial pulsatility induced by increased congestion of venous blood. In a standing position, even by asking patients to be static, movement artefacts most likely are the cause of a lower acceptance rate: it is important to highlight that under motion the device automatically aborts a measurement. In the context of the use of 24-h ABPM recordings in clinical practice, the historical consensus is that at least 70% of the expected recordings is required for an ABPM exam to be considered satisfactory^31^. While the acceptance rates reported by this study are lower than 70% for some body positions, the fact that wearable BP devices will be used for longer periods mitigates for this reduced performance: wearable BP monitors enable the first-ever persistent monitoring of BP, allowing to capture enough readings in each body position over days and weeks (as opposed to only 24 h by cuff-based ABPM monitors). It is still worth mentioning that another major difference between an ABPM device and a wearable BP monitor is that while the first requires subjects to be still during a reading to avoid measurement errors and automatically repeats failed attempts, the second doesn’t have such capability. Consequently, during long periods of movement, a wearable BP monitor will most certainly provide less BP readings than an ABPM device on a 24-h basis.

For the sitting position, this study demonstrated good accuracy of SBP and DBP readings from the Aktiia bracelet when compared to auscultatory measurements irrespectively of the position of the arm during the recording. The fact that the bias between both methods did not significantly change when lowering the wrist of the Aktiia Bracelet from the level of the heart down to the level of the lap confirms previous findings^32^ and supports the hypothesis that the BP values generated by Aktiia Bracelet are not affected by the hydrostatic drop between the heart and the wrist. Indeed, if Aktiia BP values were affected by hydrostatic gradients, the bias of this experiment would increase by + 20 mmHg (corresponding to the hydrostatic pressure drop of a standard individual between the heart and the laps). This finding is crucial for the introduction of persistent BP measurements in real-life conditions: because BP readings are not dependent on the position of the arm relative to the heart, the Aktiia Bracelet can automatically trigger measurements without the user being aware of it and removes constraints of cuff-based measurements for which the user needs to position the arm in a particularly controlled posture and then manually trigger a measurement.

For the standing and lying positions, this study demonstrated that the difference of readings between Aktiia and auscultation present larger spreads than for the sitting position. These results can be explained by the combination of two effects. First, the Aktiia Bracelet was initialized for each patient in the sitting position. By evaluating the performance on other positions, the pulse wave analysis algorithms might be prone to introduce position-dependent calibration bias. Second, the reliability of auscultatory measurements in other conditions than sitting and relaxed is not guaranteed. Although we discarded highly unlikely auscultatory measurements through a reference-control procedure, a significant source of uncertainty in the reference readings remains in this dataset. All in all, most of the assessed precisions remain below (or close to) 10 mmHg, even in such challenging real-life conditions. It is important to note that there is no international standard or consensus that covers the validation of BP monitors in such real-life conditions and that even current devices used in clinical practice for ABPM are not validated in such standing or lying body positions. In addition, it is worth highlighting that the study did not implement the additional requirements of ISO81060-2 standard^27^ for testing BP monitors intended for use in ambulatory monitoring. Unlike ABPM devices, the Aktiia Bracelet does not effectively perform measurements when the arm of the person is in motion, or while performing aerobic exercise, or not even several seconds after having stopped any physical activity: by design, the Aktiia Bracelet performs measurements only when subjects are quiet with the arm still and therefore, exercise-related validation requirements are not applicable.

As it has already been explained, out-of-office BP measurements are better predictors of cardiovascular events and are recommended by the most recent international guidelines. In particular, the nighttime measurements and the decrease in BP during the nighttime, called night dip, are known to be the best predictors of cardiovascular risk. Unfortunately, accurate out-of-office BP measurements still require the use of automated inflation cuffs, either for ABPM or HBPM procedures. The inflation of the cuff during measurement is known to trigger an increase in BP sometimes called cuff-inflation hypertension, and ABPM measurements during the night cause sleep arousal and stress, leading to higher BP readings^33^. Therefore, optical BP monitoring solutions that do not involve a cuff, hold a strong potential for 24 h BP monitoring, particularly for the nighttime measurements, and could allow better patient long term adherence to BP monitoring and reduced healthcare costs. Such devices do not involve an uncomfortable and painful cuff and could seamlessly blend into a patient’s life and accompany the patient day and night.

In this study, we assessed the performances of such a cuffless monitor, the Aktiia Bracelet, against double-blinded auscultation. In the context of addressing the feasibility of daytime and nighttime measurements, we deemed it important to go beyond assessing the performance of the device in a sitting position and we extended the recommendations of ISO 81060-2 by testing the device in other body positions. Thus, we integrated a realistic scenario of daytime and nighttime monitoring into the experimental protocol. Aware of the limitations related to Korotkoff sounds detection when performing auscultation in positions other than sitting and relaxed, we added a reference-control device to discard potentially unreliable auscultatory measurements. To test the performances of the device in a representative cohort of the intended population, we included male and female patients, with the BP values ranging from hypotensive to hypertensive as required in the ISO 81060-2 international standard. Besides, we deemed it important to address whether the quality of the optical signals recorded by the Aktiia Bracelet would compromise the reliability of the pulse wave analysis algorithms in patients with darker skin pigmentation (due to the increased absorption of the light), as well as in patients with increased hair follicle density (due to the introduction of an optical barrier). Finally, to assess the stability of the calibration, we extended the experimental protocol over 1 month. To the best of our knowledge, no other ambulatory or wearable BP monitor has disclosed results in this direction with such a complete experimental protocol and such a large and diverse cohort.

## Outlook and perspectives

This study provided an objective assessment of the accuracy that cuffless optical BP monitors can achieve when generating systolic and diastolic BP readings for the different body positions of 24/7 scenarios. It is important to note that for some body positions the differences between cuffless BP readings and simultaneous auscultatory BP readings were observed to deviate from the requirements that the ISO81060-2 defines for the sitting position. However, we hypothesize that the benefit of persistently monitoring the BP of a patient (as opposed to intermittent measurements at specified intervals) may outweigh possible deviations from an accuracy requirement that is not even tested or required under current regulations and unknown for existing devices for body positions such as standing or lying. This long-term risk–benefit ratio of such cuffless optical BP monitors is now to be assessed by means of cross-sectional and longitudinal epidemiological studies in order to test the association of persistent BP readings with target organ damage and cardiovascular events.

**Figure 8:**
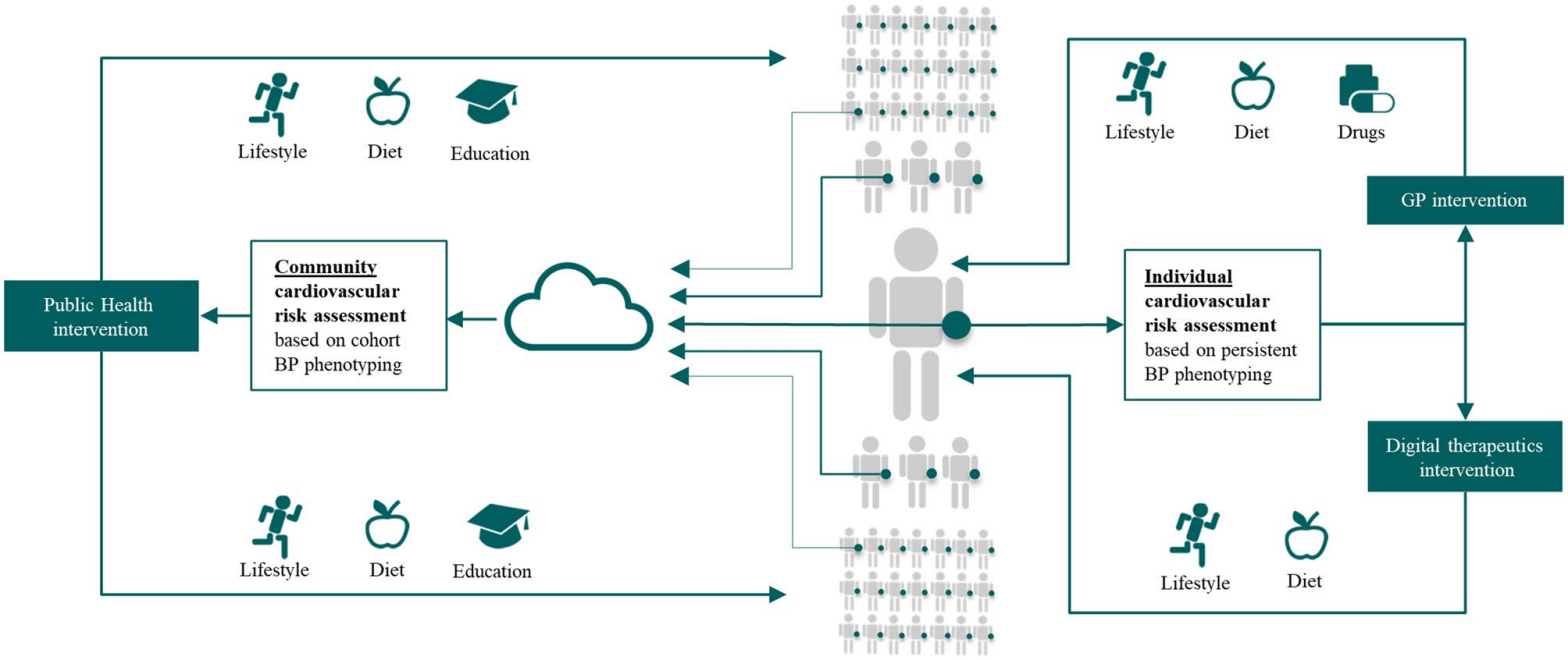
Introduction to the concept of BP phenotyping using persistent BP monitoring at the wrist, enabling a new category of studies and programs to investigate how longitudinal 24/7 measurements can improve cardiovascular outcomes at both individual and community levels.

Moreover, if accurate, the benefit of persistent BP monitoring might go beyond a simple extension of sporadic BP measurements performed on-demand. This technology enables a new category of studies and programs that can investigate how longitudinal 24/7 measurements performed at large scale support the BP phenotyping of individuals and communities. In this context, Fig. 8 provides a perspective on how persistent BP monitoring might extend and complement current existing BP monitoring modalities.

Firstly, on a patient level, the deployment of persistent BP monitoring in new clinical trials will enable to investigate how BP phenotyping services can be used to improve the assessment of individual cardiovascular risks and facilitate more targeted interventions. New digital therapeutic interventions and programs with or without GP involvement can be tested to dynamically quantify changes in individuals’ BP phenotypes at each step of titration on lifestyle or diet interventions during those trials.

Secondly, on a public-health level, the deployment of persistent BP monitoring in large-scale clinical trials will enable investigation of how community-scale cardiovascular risk scores generated by pooling BP phenotypes of entire cohorts can be used to target specific communities to improve awareness on hypertension, or intervene on salt-content of the diet of given geographical regions, or encourage lifestyle changes on a given group of age or ethnic group of a particular city for example. The implementation of such large-scale clinical trials could also be supported by different types of smartwatches and smart bands that integrate validated optical BP monitoring capabilities.

In summary, the impact of the dynamic cardiovascular risk assessment of individuals and communities that will be supported by the persistent BP monitoring at the wrist is yet to be quantified, but its potential is promising.

## Conclusion

This study has provided a first objective assessment of the performance of a wearable persistent BP monitor in different body positions. Because of its ease of use, wearable BP monitors have the potential to enable 24 h BP phenotyping of patients over long periods, and if deployed at a community level they may be also useful to drive public health interventions to detect and control hypertension.
